# Specialties

**Published:** 1883-04

**Authors:** J. B. Chandler


### SPECIALTIES.

J. B. CHANDLER.

The man who devotes himself entirely to a special branch of study must of necessity achieve a knowledge of it not attainable in any other way, yet his position is only reached by the sacrifice of a portion of the natural capabilities of his brain. This law of our physical nature equally applies to our mental powers. The perfection of physical power is only to be achieved by the uniform development of all the organs, muscles and limbs of the body. Those that are least used, or not used, soon record the loss of strength in shrinkage and ultimate paralysis.

There seems to be a link connecting all the branches of knowledge and science together in such a close union that the student who pursues one branch without reference to others, soon finds that he reaches a point beyond which he can not go without research in another, which he had at first deemed entirely without relation to his, specialty.

The rapid strides made in every branch of science have almost entirely changed methods for achieving professional and mechanical ends which where in vogue a quarter of a century ago.

Most of these changes are based upon new discoveries of the application of principles heretofore known; not the discovery of new principles. The affinities and capabilities of the original elements do not change, but man, as his wants develop, seems permitted to draw from his brain a new knowledge which enables him to meet those wants.

The brain and the elements always exist; the want and the permission develop the remedy.

From a stand point of advanced success in any science or art, we may look back upon the weak efforts of those who vainly attempted to reach success before the time had arrived when mankind absolutely required success in that direction.

Go back to the early part of this century, with comparatively a handful of people scattered over the vast area of our country, our largest cities not far exceeding in population many of the present inland towns—what use then for costly conveniences of travel, transportation, light, communication, etc.? Wonderful machines for the manufacture of goods were not required. Hand work in most instances was all sufficient.

As late as 1832 the fast passenger line between New York City and Philadelphia left Philadelphia at six o'clock in the morning, arrived at New Brunswick at eight o'clock in the evening. Passengers generally remained at New Brunswick all night and arrived at New York next morning.

As the exigencies of business and travel demanded facilities increased; a combination route of steamboat and horse power railroad at first, then the locomotive, until the time was shortened to five or six hours. Now a hundred passenger trains a day, at least, run between the two cities, many of them on two hours time.

The fastest printing presses of those days, the old "Washington" and Ramage presses, capable in first-class hands of printing eight *tokens* a day, equal to about one thousand complete newspapers of four pages, medium size, were more than sufficient for the small circulation of periodicals at that time.

Gradually the demand increased and man's brain was busy with mechanical principles. Machinery resulted which by hand power could print five hundred full copies in an hour. We have reached a point now where one newspaper requires eighty thousand copies complete, to be printed between twelve o'clock at night and six o'clock next morning.

The brain, the elements and the permission have kept pace with the requirements, and it is all easily done.

Compare the wonderful (in those days) speed of the express post boy with his relay of ponies at every ten or fifteen miles, conveying the social and business letters from one important city to another, with the telegraph and telephone or the lightning mail express of the present. With the necessity have come the means.

In this train of thought I could write pages of examples, which, when called to his attention, the reader would be familiar with. We live in the midst, daily, of such arising demands and their resulting supply.

The cultivated mind can not be contented with the mere fact of an invention, but naturally seeks for its principle and the manner of application.

The specialist may fall far short of his aim when he fails to adopt the new discovery as far as it can possibly bear upon his specialty.

Therefore, whilst not disapproving of the selection of a specialty and acknowledging the good to result therefrom, I would advocate a course of study not so absorbing in one direction as to preclude all knowledge in another.

Let your children at school be well grounded in a general knowledge of all branches and then when they find in their special vocations that a kindred science is approaching or impinging upon theirs, they will be capable of forming a judgment as to its adoption based upon a knowledge of principles upon which the advancement has been achieved.

---

A MOVEMENT AGAINST VIVISECTION.—The report of the Women's Philadelphia Branch of the Society for the Prevention of Cruelty to Animals, read at the annual meeting recently, states that the movement against vivisection is to take a more tangible form than heretofore. Arrangements are on foot for holding a public meeting, and for taking, at that meeting, the initial steps toward the formation of an Anti-Vivisection Society in Philadelphia. Other means will also be considered for the suppression of vivisection, "that deadliest of all cruelties." Meanwhile, the thousands of sick men and women who could be relieved by an application of the results obtained by vivisection are to be left to die or suffer the tortures of disease. This is the class of people who strain at a gnat and swallow a camel.—*Medical and Surgical Reporter.*

